# A comparative retrospective study on the prevalence and therapeutic treatment of dental agenesis between healthy children and children with systemic disease or congenital malformation

**DOI:** 10.1186/s12887-023-04138-3

**Published:** 2023-06-24

**Authors:** Mirja Nadolinski, Maximiliane Amelie Schlenz, Alexander Rahman, Norbert Krämer, Nelly Schulz-Weidner

**Affiliations:** 1 Dental Clinic, Department of Paediatric Dentistry, Justus Liebig University, Schlangenzahl 14, 35392 Giessen, Germany; 2 Dental Clinic, Department of Prosthodontics, Justus Liebig University, Schlangenzahl 14, 35392 Giessen, Germany; 3 Hannover Medical School, Department of Conservative Dentistry, Periodontology and Preventive Dentistry, Carl-Neuberg-Strasse 1, 30625 Hannover, Germany

**Keywords:** Dental agenesis, Hypodontia, Oligodontia, Anodontia, Systemic diseases, Congenital malformation, Paediatric dentistry, Therapeutic treatment, Oral health

## Abstract

**Background:**

Dental agenesis (DA) in the permanent dentition is one of the most common dental anomalies, with a prevalence up to 2–10%. Therefore, the aim of this retrospective study was to investigate the prevalence and therapeutic treatment of DA in healthy children (HC) compared to children with systemic disease or congenital malformation (SD/CM).

**Methods:**

Out of 3407 patients treated at the Department of Paediatric Dentistry of the Justus Liebig University Giessen (Germany) between January 2015 and December 2020, a total of 1067 patients (594 female, 473 male) aged between 4.5 and 18 years were included in this study due to DA. Besides the patients’ general medical history and therapeutic treatments, panoramic radiographs were analysed.

**Results:**

In contrast to the HC group with 9.7% DA, the SD/CM group showed a significantly higher prevalence of DA (19.8%; p < 0.05). The latter group was further classified into children with ectodermal dysplasia (4.4%), down syndrome (8.2%), cleft lip and palate (4.4%), intellectual disability/developmental delay (16.4%), and other genetic/organic diseases without intellectual disability (45.9%). Regarding therapeutic treatments, the HC group (59.5%) was significantly more often treated with an orthodontic gap opening compared to the SD/CM group (42.6%; p < 0.05), followed by orthodontic gap closing 36.5% in the HC group and 22.9% in the SD/CM group (p < 0.05), whereas no treatment was predominantly performed in the SD/CM group (37.7%) compared to the HC group (4%; p < 0.05). Furthermore, 50% in the SD/CM group required general anaesthesia for therapeutic treatment (vs. 8.1% in the HC group; p < 0.05).

**Conclusions:**

Children with SD/CM suffered more often from DA compared to HC that underlines multi- and interdisciplinary treatment of utmost importance. Furthermore, due to intellectual disability, common treatment methods can be complicated by insufficient compliance. This fact underlines the importance of an early attempt to establish the necessary cooperation enabling children with SD/CM to receive therapy.

## Background

The absence of teeth in the form of dental agenesis (DA) is the most common congenital anomaly in human dentition and can occur in both the first and permanent dentition. Depending on the number of missing teeth, hypodontia (less than 6 teeth) or oligodontia (6 or more teeth) can be differentiated from anodontia (complete absence of teeth in one dentition) [1]. In this classification, third molars are not considered.

DA can be isolated as a congenital malformation or in association with systemic disease (e.g. caused by syndromes). To date, a total of over 300 genes have been linked to tooth development [2]. In the literature, mutations of four genes in particular (Homeobox protein MSX-1, Paired box gene 9 (PAX9), axis inhibition protein 2 (AXIN2), and Ectodysplasin A (EDA) have been described as the cause underlying an absence of teeth [3].

The literature reports a diverse prevalence of approximately 2–10%, depending on the population [4]. While hypodontia has a prevalence of 6.1–10.1%, oligodontia occurs in only 0.16–0.84%, and anodontia is scarce [5]. However, if a primary tooth is missing, the likelihood of the successor not being attached is significantly increased [6]. Apart from the third molars, the lower second premolars and the upper lateral incisors are the most frequently missing [7, 8]. The exact causes are not yet fully understood. However, it can be stated that there must have been a disturbance in the early embryonic development of the ectoderm. These disturbances can be caused by environmental or genetic factors. The affected germ tissue can react in different ways, including through hypoplastic, aplastic, hyperplastic, or dysplastic activities [7].

Dental anomalies can have a negative impact on aesthetics and chewing function. The therapeutic treatment depends on the patient’s age, ability to cooperate, dentition, growth type, space available, localization, and number of missing teeth. In terms of paediatric dentistry, patients cannot be treated with fixed dental restorations such as implants or bridges until growth has been completed, which is why therapy is applied as short- to medium-term restoration in the interim.

The absence of multiple teeth, in particular, represents a psychological burden for patients. For such patients, lower self-esteem, a limited ability to communicate, and a reduced quality of life have been described [9, 10].

From a functional point of view, malocclusions, reduced chewing ability, periodontal damage, slurred speech, the lack of alveolar bone growth, and changes in the skeletal relationship can occur [9, 11]. Therefore, interdisciplinary therapy between paediatric dentistry, orthodontics, prosthodontics, oral surgery, and, if necessary, speech therapy is considered to be of great importance.

Based on the common genetic background with other developmental disorders and general systemic diseases (e.g. various forms of cancer), the most common treatment methods may be more difficult for affected patients. To the best of our knowledge, to date, the therapeutic treatment of DA in children with systemic disease has only been described in the form of case reports. Therefore, there is currently a lack of evidence on the management of DA in paediatric dentistry.

The aim of this retrospective study was to investigate the prevalence and therapeutic treatment of dental agenesis in children with systemic disease or congenital malformation (SD/CM) and compare the data to those of healthy children (HC).

The following null hypotheses were examined:

1. The prevalence of DA does not show a significant difference between the SD/CM group and the HC group.
2. There is no significant difference between the SD/CM group and the HC group concerning the number of missing teeth.
3. Therapeutic treatment of children with DA is comparable in the SD/CM group and the HC group.

## Methods

### Study groups

Data of all patients (n = 3407) treated at the Department of Paediatric Dentistry of the Justus Liebig University Giessen (Germany) between January 2015 and December 2020 were screened. A total of 1067 patients (594 female, 473 male) matched the predefined inclusion criteria (ages between 4.5 and 18 years, at least one panoramic radiograph, documentation of general medical history, and therapeutic treatments). The study protocol was approved by the Ethics Committee of the Faculty of Medicine of the Justus Liebig University Giessen (Ref. no. 28/21).

### Data collection

All relevant patient data were collected from patient files, which were screened manually in the archive. In order to comply with the legal provisions of personal data protection, patient data were transferred to a survey sheet coded with an anonymous patient identification number. The documentation included the date of first visit, age, sex, general medical history, and, if applicable, systemic diseases or congenital malformation, dental findings, and therapeutic treatment.

Medical history forms, index cards, and doctor’s letters were used to obtain the general medical background of each patient. The International Statistical Classification of Diseases and Related Health Problems (ICD-10) coding was used for coding diagnoses [12]. Children with systemic disease (SD) or congenital malformation (CM) were classified into ectodermal dysplasia (ED), down syndrome (DS), cleft lip and palate (CLP), developmental delay/intellectual disability (DD/ID), other genetic diseases without DD/ID (OGD) and other organic diseases without DD/ID (OOD).

In order to be able to make a reliable statement about dental agenesis, panoramic radiographs were used. Additionally, the tooth size was noted.

From the patient files, interdisciplinary management was verified. The therapy location was filtered out for different departments, including paediatric dentistry, orthodontics, oral surgery, or external general dentistry. Interdisciplinary orthodontic treatment measures were classified into three groups: orthodontic gap opening, orthodontic gap closing, or no treatment.

The types of care in paediatric dentistry were further differentiated into the following:

- Retainer for gap opening;
- Denture/ removable dental prostheses;
- Preservation of primary tooth, if necessary, using conservative measures;
- Extraction of primary tooth;
- Adhesive tooth reshaping;
- Auto-transplantation;
- Cantilever resin-bonded fixed dental prostheses.

### Statistical analysis

After data collection, an analysis was performed using Excel (Microsoft Office 2019, Dublin, Ireland). To maintain data protection, only previously completed survey forms were used, and the assigned patient ID number was transferred.

Statistical analysis of the data was further analysed via SPSS (version 15, IBM, Armonk, New York, USA). The chi-squared test for nominal scaled variables was used as a hypothesis test. The Man Whitney U test was performed as a non-parametric test for ordinal scaled data. The significance level was set to p < 0.05.

## Results

### Prevalence

In total, 12.9% (n = 135) of the 1067 children (759 HC and 308 children with SD/DM) suffered from DA. Among them, 12.5% (n = 59) were female, and 12.8% (n = 76) were male (p > 0.05).

Overall, 135 patients with DA had a total of 497 missing teeth. This corresponded to an average of 3.68 missing teeth per patient.

Among the HC group, 9.7% (n = 74) showed DA. A total of 19.8% (n = 61) of the children with SD/CM suffered significantly more frequently from DA than HC (p < 0.05). According to average age at first consultation, it could be detected that the HC group was older than the SD/CM group except for the DS group. Especially the children with ED were particularly young with an average age of 4.9 years (Table 1).

**Table 1:** Distribution of healthy children (HC) and children with systemic disease or congenital malformation (SD/CM, absolute [n] and relative frequency [%]) according to dental agenesis (DA) by age in years (mean and standard deviation [M(SD)]) of first consultation

	HC group		SD/CM group												
	**% (n)**	**age** **M** (**SD**)	**ED** **% (n)**		**age** **M** (**SD**)	**DS** **% (n)**	**age** **M** (**SD**)	**CLP** **% (n)**	**age** **M** (**SD**)	**DD/ID** **% (n)**	**age** **M** (**SD**)	**OGD** **% (n)**	**age** **M** (**SD**)	**OOD** **% (n)**	**age** **M** (**SD**)
**DA**	9.7 (n = 74)	9.8 (3.7)	4.4 (n = 6)		4.9 (2.1)	8.2 (n = 11)	10.7 (1.4)	4.4 (n = 6)	7.7 (2.1)	7.4 (n = 10)	8,5 (0.0)	7.4 (n = 10)	7.9 (1.6)	13.3 (n = 18)	8.0 (2.8)

ED = ectodermal dysplasia, DS = Down syndrome, CLP = cleft lip and palate, DD/ID = developmental delay/intellectual disablility, OGD = other genetic diseases without DD/ID, OOD = other organic diseases without DD/ID.

HC showed less hypodontia (8.3%), oligodontia (1.4%), and anodontia (0%) than children with SD/CM (Table 2).

**Table 2:** Relative [%] and absolute [n] frequency of missing teeth severity based on patient population with dental agenesis distributed to healthy children (HC) and children with systemic disease or congenital malformation (SD/CM)

Severity	HC group in % (n)	SD/CM group in % (n)
Hypodontia	8.3% (n = 63)	15.9% (n = 49)
Oligodontia	1.4% (n = 11)	3.6% (n = 11)
Anodontia	0 (n = 0)	0.3% (n = 1)

There could be demonstrated a difference in the severity of DA based on the patient population. Whereas HC mostly presented hypodontia, the distribution of severity within the SD/DM group was as follows. While 83.3% of patients presenting ED had oligodontia and 16.7% had anodontia, hypodontia was more common in the other groups (Table 3).

**Table 3:** Relative [%] and absolute [n] frequency of severity of hypodontia, oligodontia and anodontia in healthy children (HC) and children with systemic disease or congenital malformation (SD/CM)

	HC group in % (n)	SD/CM group in % (n)					
		ED	DS	CLP	DD/ID	OGD	OOD
**Hypodontia**	85.0 (n = 63)	0 (n = 0)	81.8 (n = 9)	100 (n = 6)	90.0 (n = 9)	90.0 (n = 9)	88.9 (n = 16)
**Oligodontia**	14.9 (n = 11)	83.3 (n = 5)	18.2 (n = 2)	0 (n = 0)	10.0 (n = 1)	10.0 (n = 1)	11.1 (n = 2)
**Anodontia**	0 (n = 0)	16.7 (n = 1)	0 (n = 0)	0 (n = 0)	0 (n = 0)	0 (n = 0)	0 (n = 0)

ED = ectodermal dysplasia, DS = Down syndrome, CLP = cleft lip and palate, DD/ID = developmental delay/intellectual disability, OGD = other genetic diseases without DD/ID, OOD = other organic diseases without DD/ID.

Of the patients with missing teeth, more teeth were missing in the mandible (54.3%) than in the maxilla (45.7%). Overall, the following teeth were missing in descending order: lower second premolars (24.4%), upper second incisors (13.7%), and upper second premolars (10.7%).

Analysis of the individual groups provided the following results. While HC patients showed the same rankings of missing tooth groups as the total patient population, the lower (42.6%) and upper (18%) second premolars were more frequently missing in developmentally delayed patients. In patients with DS, mainly the upper second incisors were missing (28.6%), followed by the lower second premolars (20.4%). The clinical picture of ED showed an almost even distribution of non-apposed teeth, whereas in CLP, upper and lower incisors (45.4%) or premolars (45.4%) were affected equally.

In addition, out of a total of 1067 patients, conical teeth were diagnosed in 20 patients. Among them, 60% of the HC group and 40% of children with SD/CM suffered from so-called conical teeth.

### Therapeutic treatments

In total, 48.9% of patients with missing teeth were exclusively treated by paediatric dentists. Interdisciplinary treatment with orthodontists occurred in 23% of cases. Children with SD/CM were presented to orthodontists less frequently. Overall, orthodontic gap opening was chosen as the treatment approach in 50.4% of cases, followed by orthodontic gap closure (30.3%) and “no therapy” (19.3%). Of these, children with SD/CM were less often treated with interdisciplinary gap closure (22.9% vs. 36.5% in HC) or gap opening (42.6% vs. 59.5% in HC). In 37.7% of children with SD/CM, no orthodontic therapy could be arranged. In contrast, only 4.0% of HC decided not to start orthodontic treatment.

Table 4 outlines the various orthodontic therapy decisions and corresponding percentages. Especially in patients with DS and CLP, no orthodontic treatment was selected, representing the majority of therapy.

**Table 4:** Relative frequency [%] and absolute frequency [n] of therapy approaches for healthy children (HC) and children with systemic disease or congenital malformation (SD/CM)

Therapeutic treatment	HC group in % (n)	SD/CM group in %(n)					
		**ED**	**DS**	**CLP**	**DD/ID**	**OGD**	**OOD**
Orthodontic gap opening	59.2 (n = 44)	100 (n = 6)	18.2 (n = 2)	16.7 (n = 1)	40.0 (n = 4)	40.0 (n = 4)	27.8 (n = 7)
Orthodontic gap closing	36.9 (n = 27)	0 (n = 0)	9.1 (n = 1)	16.7 (n = 1)	10.0 (n = 1)	20.0 (n = 2)	50.0 (n = 9)
No treatment	3.9 (n = 3)	0 (n = 0)	72.7 (n = 8)	66.7 (n = 4)	50.0 (n = 5)	40.0 (n = 4)	11.1 (n = 2)

ED = ectodermal dysplasia, DS = Down syndrome, CLP = cleft lip and palate, DD = developmental delay/intellectual disability, OGD = other genetic diseases without DD/ID, OOD = other organic diseases without DD/ID.

Regarding paediatric dental therapy, the following types of care were reported among children with DA: preservation of primary teeth (52.6%), extraction of primary teeth (27.4%), cantilever resin-bonded fixed dental prostheses (6.7%), paediatric dentures (3.7%), adhesive reshaping of teeth (3.7%), and auto- transplantation (0.75%) (Table 5).

**Table 5:** Relative frequency [%] and absolute frequency [n] of measurements in healthy children (HC) and children with systemic disease or congenital malformation (SD/CM)

Therapeutic treatment	HC group in % (n)	SD/CM group in % (n)					
		**ED**	**DS**	**CLP**	**DD/ID**	**OGD**	**OOD**
Retainer for gap opening	0 (n = 0)	0 (n = 0)	0 (n = 0)	0 (n = 0)	0 (n = 0)	0 (n = 0)	0 (n = 0)
Dentures/ removable dental prosthesis	0 (n = 0)	83.3 (n = 5)	0 (n = 0)	0 (n = 0)	0 (n = 0)	0 (n = 0)	0 (n = 0)
Preservation of primary tooth	60 (n = 44)	0 (n = 0)	66.7 (n = 7)	33.3 (n = 2)	40.0 (n = 4)	40.0 (n = 4)	66.7 (n = 12)
Extraction of primary tooth	20 (n = 15)	0 (n = 0)	33.3 (n = 4)	66.7 (n = 4)	20.0 (n = 2)	30.0 (n = 3)	66.7 (n = 12)
Adhesive tooth reshaping	5.5 (n = 4)	16.7 (n = 1)	0 (n = 0)	0 (n = 0)	0 (n = 0)	0 (n = 0)	0 (n = 0)
Auto-transplantation	1.8 (n = 1)	0 (n = 0)	0 (n = 0)	0 (n = 0)	0 (n = 0)	0 (n = 0)	0 (n = 0)
Cantilever resin-bonded fixed dental prostheses	12.7 (n = 9)	0 (n = 0)	0 (n = 0)	0 (n = 0)	0 (n = 0)	0 (n = 0)	0 (n = 0)

ED = ectodermal dysplasia, DS = Down syndrome, CLP = cleft lip and palate, DD = developmental delay/intellectual disability, OGD = other genetic diseases without DD/ID, OOD = other organic diseases without DD/ID.

Patients with ED were mainly restored with paediatric dentures or reshaping on adjacent teeth was conducted. Cantilever resin-bonded fixed dental prostheses and auto-transplantation were exclusively performed in patients without SD/CM.

Moreover, for required primary tooth restorations and primary tooth extractions, dental treatment under general anaesthesia was performed in 50% (n = 16) of children with DA associated with SD/CM. HC group, on the other hand, could be treated chairside in 91.9% of cases (n = 37).

## Discussion

In the present study, a selected patient population was examined in the period from January 2015 to December 2020. Thus, a period of six years was considered, enabling a comparison with similar studies in the literature [9, 13].

The American Dental Association [14] has established guidelines for radiographs, recommending panoramic radiographs to ensure the growth and development of dentofacial structures. Based on these considerations, the present study was limited to existing panoramic radiographs. The justifying indications for this included the assessment of tooth development or tooth germs, tooth malpositions, structural disorders of the tooth structure, or trauma. Thus, no new radiological findings were made, which would have entailed additional radiation exposure for the patients and would not have been ethically justified. Assessment of missing teeth in the permanent dentition could be retrospectively misdiagnosed if teeth were lost due to caries, trauma, or extraction caused by an orthodontic indication. These possible sources of error could be reduced by using several panoramic radiographs over a period of time along with clinical photographs.

Based on the data collected at a dental clinic, it is possible that the study sample features more missing permanent tooth structures or teeth than the general population. There is also a potential tendency for a higher percentage of oligodontia and anodontia due to the patient population attending a university dental facility. The study population may also be somewhat skewed because dentists in private practice often refer patients with complex cases to the university for care. However, the panoramic shift sample included over 60% of children not taking any medication, suggesting that referrals were due to children’s behavioural problems rather than the complexity of the dental procedures.

A strength of this study was the availability of up-to-date panoramic radiographs. The sample size provided an adequate representation of the patient population at the Department of Paediatric Dentistry. In most cases, parents with children suffering from systemic disease or a congenital malformation visit a highly specialised dental clinic or are referred directly by the general dental practice. The close cooperation with the in-house polyclinic for orthodontics additionally facilitated the documentation of therapy approaches.

Within the patient population, 12.9% were found to suffer from DA. Depending on the study design and population group, a prevalence range of 1.6–12.6% has been described in the literature [9, 13]. Thus, our results are slightly higher than the upper range in previous literature. The wide range in the prevalence of non-congenital cases could be attributed to differences in sampling methods and sample size, as well as variations in origin [15]. In addition, most studies excluded children and adolescents with general diseases. The available data from healthy patients with missing teeth showed a frequency of 9.7%, which is in agreement with other authors [13, 16].

In addition, some authors do not present the severity of missing teeth and thus hypodontia, oligodontia, and anodontia are not differentiated more precisely [16]. With regard to the subdivision of the severity of DA, our study was consistent with a previous study by Behr et al. [9] that reported 12% hypodontia and 2% oligodontia in children at a University Hospital (our results were 10.5% hypodontia, 2.1% oligodontia, and 0.1% anodontia). In comparison, children with systemic diseases more often presented missing teeth in the form of hypodontia (15.9%), oligodontia (3.7%), or anodontia (0.3%). Due to the scarcity of studies on children with systemic diseases, no comparison can be made.

Looking at the different patient groups, patients with ectodermal dysplasia (6 out of 6 patients), Down syndrome (11 out of 14 patients), or cleft lip and palate (6 out of 8 patients) showed more missing teeth. ED, in particular, was associated with oligodontia (83.3%) or anodontia (16.7%), as confirmed by other studies showing an association of these diseases with hypodontia [17, 18]. Patients with DS commonly suffer from congenital heart defects. Reuland-Bosma et al. [19] also found that congenital heart defects and hypothyroidism in children with DS are parameters for DA.

One limitation here is that no calibration took place due to the retrospective data collection. However, since the practitioners underwent the same training in their studies, as well as postgraduate training at the department, this factor is likely negligible.

Our results showed that agenesis of the second lower premolars was most common, followed by agenesis of the upper lateral incisors, upper second premolars, and lower central incisors. Most previous studies have confirmed these results [15, 20]. In a sample of 5127 patients, agenesis of the maxillary lateral incisors occurred with a frequency of 2.2% and agenesis of the second premolar with a frequency of 3.4% [21]. In turn, other authors highlighted the maxillary lateral incisor as the most frequently affected tooth [22]. It was also observed that the terminal tooth in a row of teeth (incisors, premolars, and molars) is the most frequently missing [23]. In the evolutionary process, these teeth did not provide a selective advantage to the species and were, therefore, lost [24]. Kaer [25] described the location of tooth agenesis based on neural developmental fields in the jaw (incisor field, canine/premolar, and molar field). The region within a single field where innervation occurs last was more likely to show DA. However, these data were limited to children and adolescents without general diseases.

Among patients with DS, primarily the second upper incisors were absent in the present study. This was also confirmed through a meta-analysis by Palaska et al. [17], who investigated the frequency of missing teeth in children with DS. Russel et al. [26] also reported that children with DS were ten times more likely to suffer from missing teeth compared to healthy children.

ED showed a relatively even distribution of affected missing teeth. However, due to the rarity of ED, the number of cases was not very large and involved only six patients. A comparison with the literature is also difficult because there are few studies on the missing teeth patterns of affected children; mostly, such studies are individual case reports.

Patients with CLP were affected by agenesis of the incisors and premolars in both the maxilla and mandible. Again, the case number of six patients is very small and should be confirmed by further studies. Due to the cleft formation in the upper jaw, malformations and missing teeth can certainly occur in regions of the incisors [27].

As in the patients without SD/CM, the lower second premolar was the most frequently found missing in developmentally delayed patients, followed by the second upper premolar. Currently, there are no studies reporting missing teeth in children with developmental delays.

Therapeutic approaches are described in the literature mainly from an orthodontic perspective. Therefore, a comparison with paediatric dentistry is hardly possible. Our results clarified that affected patients are not exclusively treated in orthodontic practice but that therapeutic measures in paediatric dentistry are also important. Among the total patient population, 71.9% underwent therapy through paediatric dentistry. The present study, therefore, focused on the care measures of paediatric dentistry for DA. Due to the age, no therapy for definitive prosthetic measures or implant therapy could be considered.

For up to 37.7% of the affected patients, no orthodontic therapy was possible. In healthy patients, only 4% could not be treated with orthodontics. Many children with general illness have cognitive limitations, and the resulting lack of cooperation makes chairside treatment and thus orthodontic therapy impossible. Therefore, mainly children with DS were affected by the therapeutic decision of “no treatment”. The mental abilities of people with DS vary widely. Thus, the literature contains rare information about the prosthetic rehabilitation of patients with DS and tends to consist of individual case reports [28]. In addition, the patient’s cooperation may be reduced and there may be an increased risk of root resorption during orthodontic therapy [29]. Early involvement of patients in dental care with DS/ID could possibly lead to achieving habituation and cooperation for necessary dental therapy and in addition, in order to achieve a fast and therapeutically satisfactory treatment result orthodontic treatment should be carried out simple and realistic.

The types of restorations in paediatric dentistry were mainly a decision between “primary tooth preservation” and “primary tooth extraction” in diseased children. More complex types of restoration, such as cantilever resin-bonded fixed dental prostheses, were performed exclusively in healthy patients. Only children with ED could be fitted with paediatric dentures. This result is also related to the young age of the children presented firstly with an average of 4.9 years. Due to the multiple DA, functional and esthetic rehabilitation should be performed early and in appropriate cooperation, which is only possible through the use of paediatric dentures which is why these results of therapy seem unsurprising. The prosthetic rehabilitation of these patients with paediatric prostheses was confirmed by individual case reports [30, 31]. The limitations mentioned above also apply here, as the known distribution patterns of non-unions in, e.g., ectodermal dysplasia may have led to a distortion of the therapeutic approaches.

## Conclusions

Children with SD/CM suffered more often from DA compared to HC. Regarding the most frequent therapy, orthodontics were predominantly performed in HC showing the need of early referral to an orthodontist so that children with SD/CM can also receive necessary orthodontic therapy in case of appropriate compliance. Multi- and interdisciplinary treatment has to be of utmost importance in order of an attempt to establish the necessary cooperation enabling children with SD/CM to receive therapy.

## Data Availability

The datasets in this article are available from the corresponding author upon reasonable request.

## Abbreviations

AXIN2: Axis inhibition protein 2
CLP: Cleft lip and palate
CM: Congenital malformation
DA: Dental agenesis
DD: Developmental delay
DS: Down Syndrome
ED: Ectodermal Dysplasia
EDA: Ectodysplasin A
HC: Healthy children
ID: Intellectual Disability
MSX-1: Homeobox protein
OGD: Other genetic diseases without DD/ID
OOD: Other organic diseases without DD/ID
PAX9: Paired box gene 9
SD: Systemic disease
